# A rapid method of evaluating cytotoxic drug efficacy using sub-cellular fluctuation imaging

**DOI:** 10.1038/s41598-025-30295-9

**Published:** 2025-12-06

**Authors:** Henrik Rehnstrom, Arthur Eley, Natasha S. Clayton, Georgina Plant, Diego Oviedo-Chavez, Ben Ede, Darryl J. Hill, Anne J. Ridley, Massimo Antognozzi

**Affiliations:** 1 School of Physics, University of Bristol, Tyndall Avenue, Bristol, BS8 1TL UK; 2 School of Cellular and Molecular Medicine, University of Bristol, University Walk, Bristol, BS8 1TD UK; 3 Department of Physics, University of Oxford, Parks Road, Oxford, OX1 3PU UK

**Keywords:** SCFI, Sub-cellular fluctuation imaging, Cancer, Assay, Cytotoxic, Antineoplastic, Testing, Staurosporine, Paclitaxel, PC3, PC-3, Caco-2, Calu-3, A549, Microscopy, Cellular imaging, Biophysics, Cancer, Cell biology, Biological physics, Techniques and instrumentation

## Abstract

Determining whether potential cancer therapies effectively kill cancer cells is important for informing effective therapeutic choice for patients. Here we describe a rapid label-free method for testing drug efficacy in vitro that evaluates cellular viability from sub-cellular fluctuation imaging (SCFI). We used staurosporine and paclitaxel as known cytotoxic drugs at different concentrations, and four different human cancer-derived cell lines: PC3 (prostate), Caco-2 (colorectal), Calu-3 (lung) and A549 (lung). Both drugs caused a rapid decrease in sub-cellular fluctuations within 1 to 3 h except when the specific cell line was known to be resistant to one of the drugs. We also demonstrated that the method is able to differentiate between treated and untreated PC3 cells within 3 to 4 h after cells have been plated, thus eliminating the need for overnight incubation, and further decreasing the total time needed to evaluate drug efficacy. SCFI is therefore able to identify reliably if drugs are cytotoxic within 3 h of addition, which is considerably faster than current commonly used techniques.

## Introduction

Conventional methods of testing antineoplastic drug efficacy in vitro vary in reliability (when compared to in vivo testing) and are relatively slow^1–3^. Common colorimetric assays (such as MTT, lactate dehydrogenase/LDH and neutral red) usually take several days to produce reliable results^4^. Alternative assays, such as those based on fluorescein isothiocyanate or Hoechst staining are faster, but remain dependent on molecular labels, which could affect measurements^5–8^. Recent improvements in high-throughput drug screening have increased both the reliability and speed of testing; however, the methods still require a relatively expensive setup, and can only become cost effective when high numbers of drugs are tested^3,9^. There is therefore a need for a quick, reliable and label-free method to test the efficacy of potential cancer therapies on a smaller scale in vitro.

Since the discovery in 2004 that living cells exhibit characteristic nanoscale fluctuations, which end upon cell death^10^, this has been the basis for several methods used to evaluate cellular viability^11–21^. So far, the main use of this discovery has been Antimicrobial Susceptibility Testing (AST), which often take days to perform using current growth-based methods^22,23^. Fluctuation-based AST was first undertaken with an atomic force microscopy cantilever upon which groups of bacteria were attached^12–14^. Even though this method could only measure the mean cellular fluctuation of many bacteria, it was still able to reliably differentiate living and dead cells^12^. A variation of this method reduced environmental effects on cell viability by using a bimaterial cantilever with an embedded microfluidic channel^15^. Plasmonic imaging has also been successfully used to evaluate bacterial cellular viability from fluctuations^16,17^, as have a number of electronic and acoustic methods of fluctuation-based AST^18–20^.

The nanoscale fluctuations measured may derive from cellular metabolism (and/or possibly from cytoskeletal dynamics)^10,21,24^. If so, then this means that the measurement of sub-cellular fluctuations could have several advantages when compared to measuring the fluctuations of entire, or even multiple, cells; the latter being the basis for all the above methods. To address this, we have developed a new method called Sub-Cellular Fluctuation Imaging (SCFI), which measures fluctuations inside individual cells in real time^21^. This method reliably detects the effects of anti-microbial treatments on baceria in as little as minutes, and experimental SCFI data on bacteria also supports the hypothesis that the fluctuations may be related to metabolism as they are able to identify different bacterial metabolic states from fluctuation analysis alone^21^. Further research on the relationship between the fluctuations and metabolism is however needed before any definitive conclusions can be drawn, as alternative causes of nanoscale fluctuations could be influenced by metabolic inhibitors and/or growth rate. If the fluctuations are related directly or indirectly to metabolic processes, then they should presumably also be present in all living cells, whether prokaryotic or eukaryotic^11^.

Here, we demonstrate that SCFI can be used to evaluate the viability of human cancer cells, using several different cell lines (PC3, Caco-2, Calu-3 and A549) and two different treatments known to reduce viability in the long term (staurosporine and paclitaxel). We also show that SCFI can be used to evaluate if an antineoplastic drug is able to affect the targeted cells effectively, or if a given cell line is resistant to a particular treatment.

**Fig. 1:**
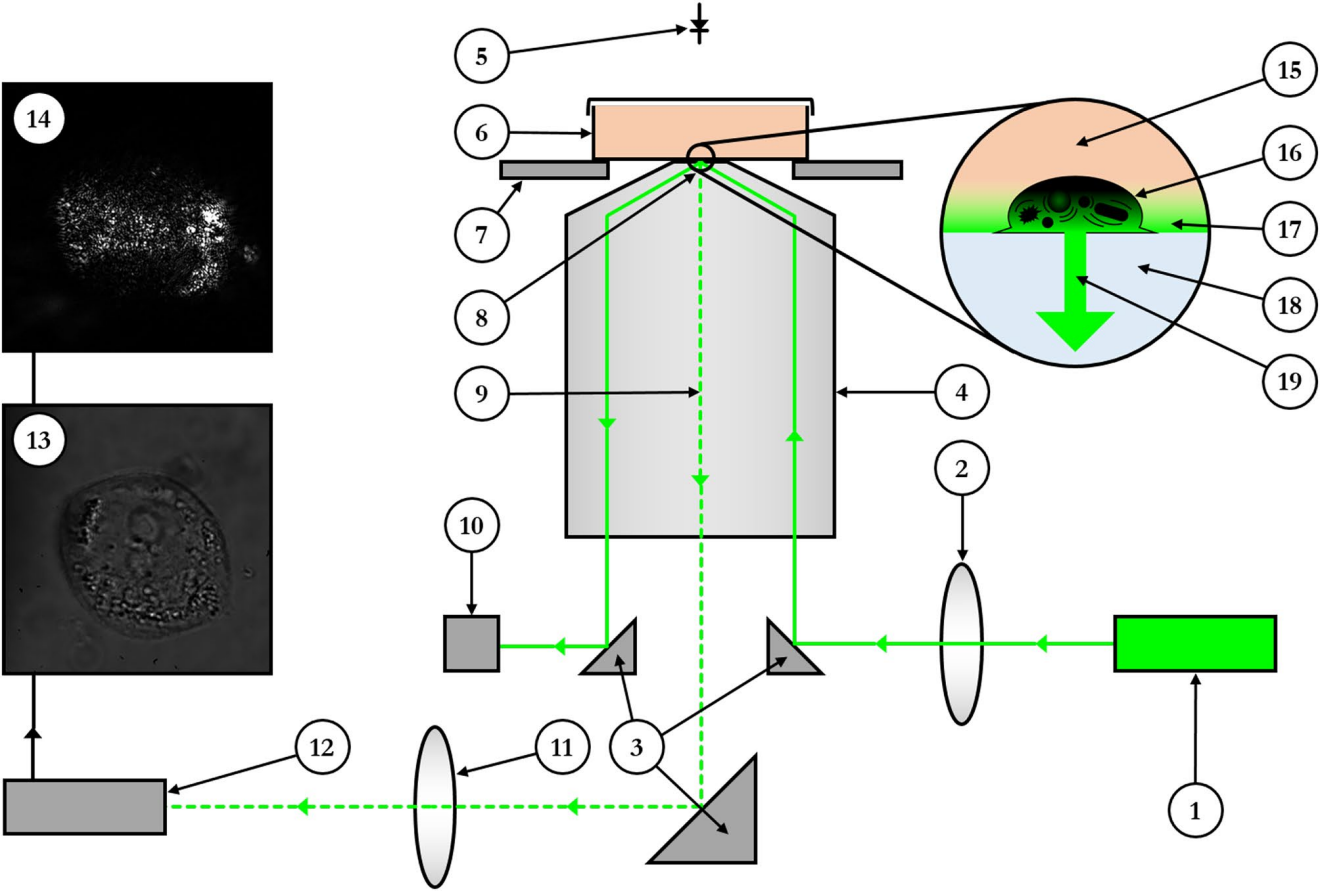
**Schematic of the Sub-Cellular Fluctuation Imaging (SCFI) system.** The setup is based on an objective-type total internal reflection (TIR) microscope. A high numerical aperture objective (4) directs a collimated laser beam (1) at the correct angle to generate an evanescent field (17) at the surface of the sample dish (6). Light scattered (9, 19) by subcellular components within a cell (16) is collected by the same objective and imaged onto a CMOS camera (12), producing a characteristic scattering pattern (14). The system is also equipped for conventional bright-field imaging (13) using an LED (5). Numbered components are as follows: (1) laser source, (2) lens used to collimate the laser beam after the objective (4), (3) right-angle mirrors, (4) high NA objective, (5) LED illuminator, (6) Petri dish with sample, (7) x-y positioning stage, (8) TIR of laser beam, (9) scattered light path, (10) beam block, (11) tube lens, (12) CMOS camera, (13) bright-field image example, (14) scattering pattern example, (15) cell culture medium, (16) cell, (17) evanescent field, (18) dish substrate, (19) scattered light from cell

The optical system used in SCFI (Fig. 1) is based upon the Scattered Evanescent Wave (SEW) detection system developed to measure the tip of micro-cantilever deflection near a surface. The SEW method combined objective-based total internal reflection (TIR) with evanescent scattered microscopy (ESM) increasing resolution through homedyne interference^25,26^. SCFI is a further refinement of the SEW method in which the optical sensitivity is enhanced by combining the evanescent field illumination with the interference caused by the optical interface. In SCFI, the total internal reflection of the laser beam is generated by a high numerical aperture objective lens, and a video camera subsequently collects the light scattered by samples illuminated by the evanescent field created above the TIR boundary. The optical system is also capable of collecting conventional bright field images of the sample. Real-time recordings of the scattered light observed in SCFI reveal intensity fluctuations over time if any part of the sample is moving within the evanescent field (e.g. movement of sub-cellular organelles).

**Fig. 2:**
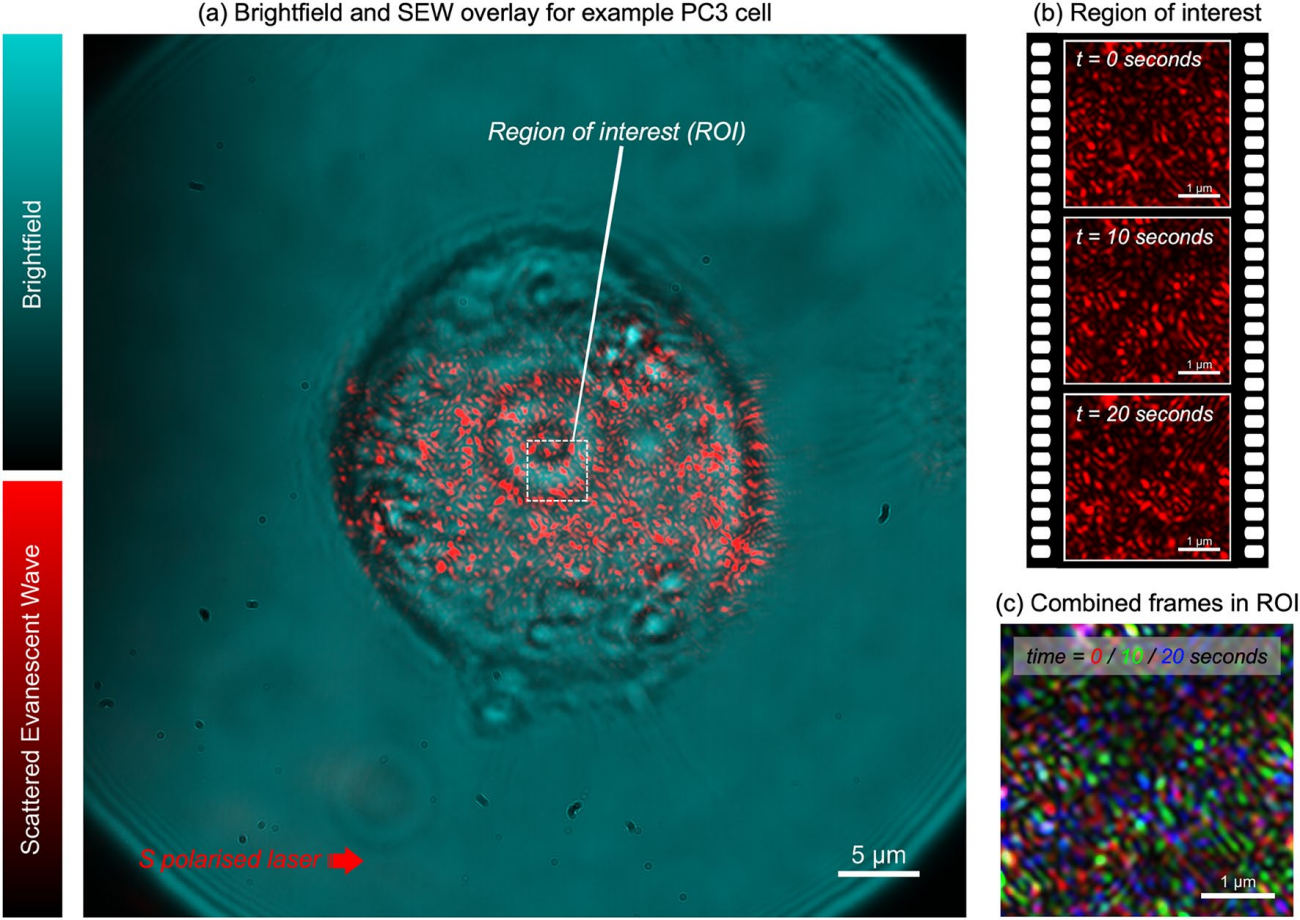
**Visualising subcellular fluctuations in a PC3 prostate cancer cell with SCFI.** **(a)** A composite image displays a standard bright-field view of a PC3 cell (cyan) overlaid with the scattered evanescent wave (SEW) signal (red), originating from the cell’s internal components near the glass surface. The analysis is performed on a small region of interest (ROI) at the centre of the cell. Scale bar: 5 $\upmu$m. **(b)** A time-series of raw SEW images captured from within the ROI at 0, 10, and 20 seconds. The speckled pattern varies over time, indicating the continuous movement of subcellular structures. Scale bar: 1 $\upmu$m. **(c)** A false-colour overlay of the three frames from (b), where *t* = 0 s is red, *t* = 10 s is green, and *t* = 20 s is blue. Static components present in all three frames appear white, while components that have moved or changed between frames show as distinct colours (red, green, or blue). This colour separation visually illustrates the subcellular fluctuations that are quantified by the autocorrelation analysis

In SCFI, short videos are taken of the light scattered by the internal components of a single cell (Fig. 2). These videos are then analysed to measure the speed and magnitude of the subcellular movements. This is done using a technique called time autocorrelation function (ACF) analysis, which essentially measures how quickly the image of the cell changes over time^27–29^. From this analysis, we extract two key metrics: the fluctuation amplitude ($g_{0}$), which represents the magnitude of the movements, and the fluctuation time constant ($\tau _{D}$), which represents their speed. A decrease in the amplitude and an increase in the time constant (slower movement) are indicators of reduced cellular activity and, ultimately, cell death^21^. A more detailed description of the analysis is provided in the “Methods” section.

## Results

### Effects of cytotoxic drugs on PC3 cells detected using SCFI

To determine whether SCFI could be used to detect the effects of chemical inhibitors in human cells, as already observed in bacterial cells^21^, PC3 prostate cancer cells were tested for responses to the cytotoxic drugs staurosporine (8 $\upmu$M) and paclitaxel (10 $\upmu$M) or DMSO as control. The concentrations used were based on a combination of previous literature and laboratory practice^30–32^. All samples were prepared under identical conditions (apart from the addition of drug or control vehicle; see the “Methods” section, “Sample preparation” subsection, for details), and PC3 cells were plated the day before SCFI measurements.

**Fig. 3:**
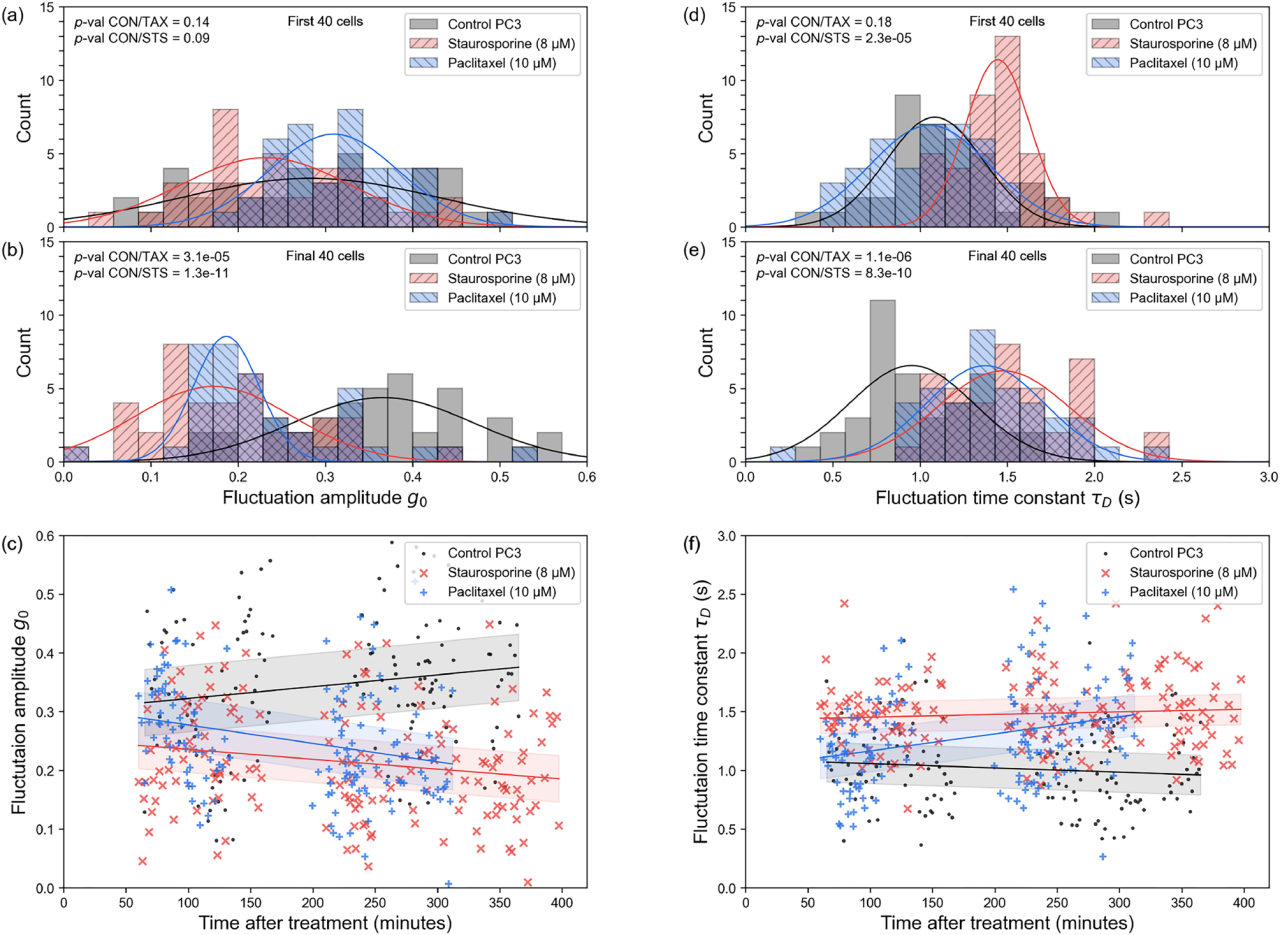
**Staurosporine and paclitaxel induce a time-dependent reduction in subcellular fluctuations in PC3 cells.** SCFI measurements were performed on PC3 prostate cancer cells treated with either staurosporine (8 $\upmu$M), paclitaxel (10 $\upmu$M), or a vehicle control (DMSO). **(a, d)** Histograms of fluctuation amplitude ($g_{0}$) and time constant ($\tau _{D}$) from the first 40 cells measured in each group, showing no significant difference between treated and control cells at early time points (within approximately 1.5 h). **(b, e)** Histograms from the final 40 cells measured (after approximately 3 h), showing a clear and statistically significant decrease in fluctuation amplitude and an increase in fluctuation time constant for drug-treated cells, indicating reduced cellular activity (see “Methods”). **(c, f)** Scatter plots showing the evolution of $g_{0}$ and $\tau _{D}$ for every individual cell measured over the entire $\approx$6-h experiment. Each point represents a single cell. The linear trendlines (with 95% confidence bands) show that for the control cells (grey), activity remains stable, whereas for the drug-treated cells (red and blue), activity progressively decreases over time. Data were pooled from three independent experiments (total n = 450 cells, of which 140 are control, 160 are staurosporine treated, and 150 are paclitaxel treated). See Table 1 for more data statistics

**Table 1:** Detailed statistics for data presented in Fig. 3b, e. Means, standard deviations and limits. CON, Control; STS, staurosporine; TAX, paclitaxel

PC3	$g_{0}$ mean	$g_{0}$ SD	$g_{0}$ lower	$g_{0}$ upper	$\tau _{D}$ mean	$\tau _{D}$ SD	$\tau _{D}$ lower	$\tau _{D}$ upper
CON	0.36	0.11	0.33	0.40	0.99	0.33	0.89	1.09
STS	0.19	0.09	0.16	0.22	1.51	0.35	1.40	1.62
TAX	0.25	0.13	0.21	0.29	1.40	0.38	1.28	1.52

Using either the fluctuation amplitude or the fluctuation time constant for all cells for each treatment condition, the SCFI method was clearly able to differentiate between treated and untreated cells within 4 h of exposing the cells to each cytotoxic drug (Fig. 3). Given that the concentrations of staurosporine and paclitaxel used here have previously been reported to kill PC3 cells, these results are consistent with the previous literature, including the relative resistance to staurosporine in PC3 cells^30–32^. This shows that SCFI can rapidly detect the effects of well-characterised cytotoxic drugs (including paclitaxel, a drug used for over 40 years in clinical oncology)^33^.

Initial variation between cell fluctuations (before 180 min) likely stems from temperature differences during the transport of samples between laboratories. The duration of this journey averaged approximately 20 min. The changing of medium from RPMI to L-15 (which is buffered to air $\text{ CO}_2$, and necessary due to unavailability of incubator with $\text{ CO}_2$ control where the above SCFI measurements were acquired) does not seem to be a cause of this variation; a preliminary test comparing PC3 cells in RPMI and L-15 without any outdoor transportation (using a different SCFI microscope) showed no statistical difference between the two (Figure S1 in Supplementary Information).

### Effects of staurosporine and paclitaxel on Caco-2 cells detected using SCFI

**Fig. 4:**
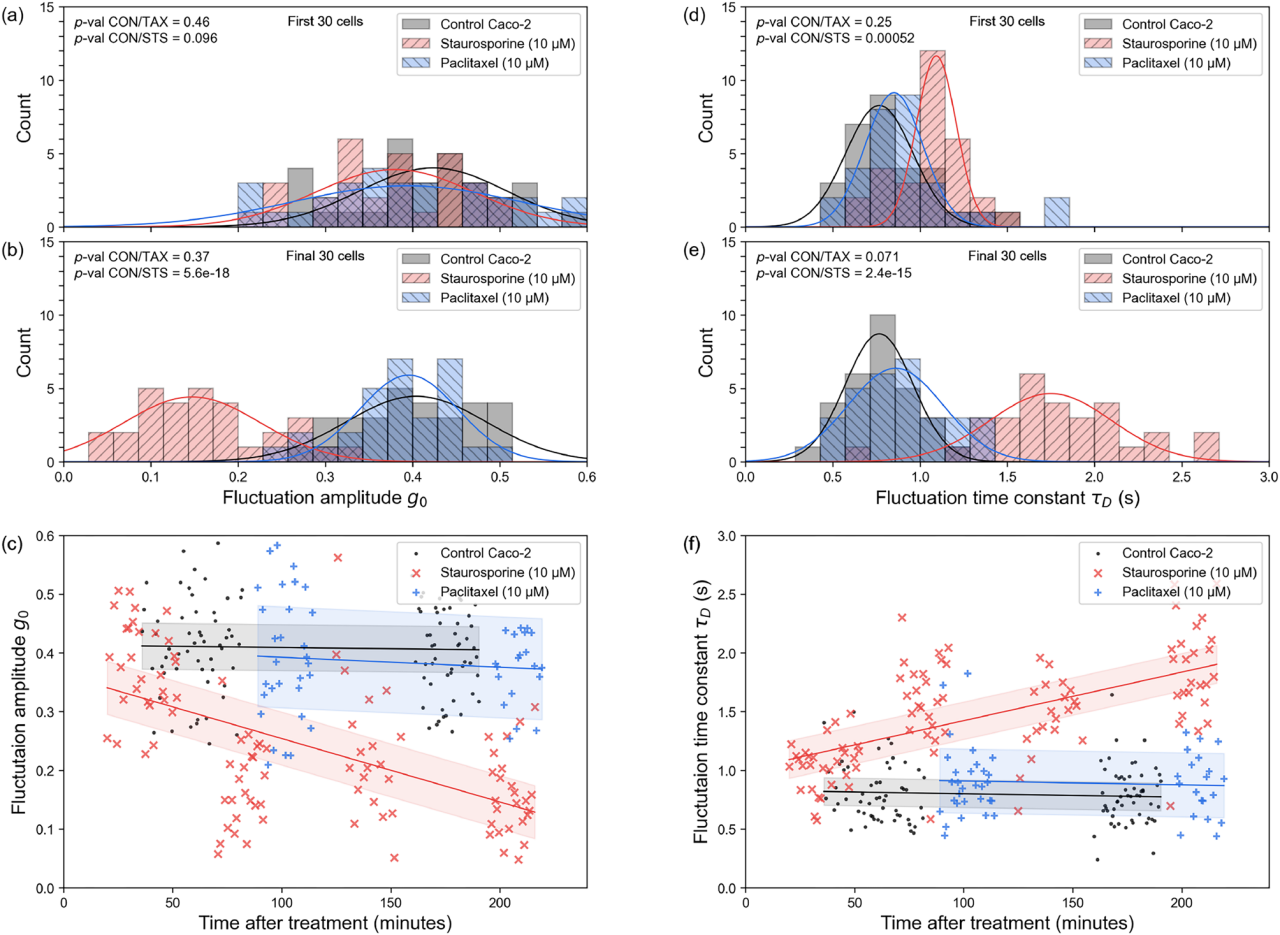
**SCFI detects a cytotoxic response to staurosporine but not paclitaxel in Caco-2 cells, demonstrating drug-specific sensitivity.** Caco-2 colorectal cancer cells, known to be resistant to paclitaxel, were treated with either staurosporine (10 $\upmu$M), paclitaxel (10 $\upmu$M), or a vehicle control (DMSO). **(a, d)** Histograms of fluctuation amplitude ($g_{0}$) and time constant ($\tau _{D}$) from the first 30 cells measured show no significant differences between the groups at early time points. **(b, e)** Histograms from the final 30 cells measured (after approximately 3 h) reveal a significant response to staurosporine (red), but no significant change in cells treated with paclitaxel (blue) compared to the control (grey). **(c, f)** Scatter plots of $g_{0}$ and $\tau _{D}$ over the entire experiment confirm the time-dependent effect. The linear trendlines (with 95% confidence bands) show that the activity of staurosporine-treated cells progressively decreases, while the activity of paclitaxel-treated cells remains indistinguishable from the control. This result confirms that SCFI can distinguish between effective and ineffective drug treatments for a specific cell line. Data were pooled from three independent experiments (total n = 285 cells, of which 110 are control, 100 are staurosporine treated, and 75 are paclitaxel treated). See Table 2 for more data statistics

**Table 2:** Detailed statistics for data presented in Fig. 4b, e. Means, standard deviations and limits. CON, control; STS, staurosporine; TAX, paclitaxel

Caco-2	$g_{0}$ mean	$g_{0}$ SD	$g_{0}$ lower	$g_{0}$ upper	$\tau _{D}$ mean	$\tau _{D}$ SD	$\tau _{D}$ lower	$\tau _{D}$ upper
CON	0.40	0.07	0.37	0.43	0.78	0.20	0.70	0.85
STS	0.16	0.08	0.13	0.19	1.79	0.41	1.63	1.94
TAX	0.38	0.06	0.36	0.41	0.89	0.27	0.79	0.99

To investigate whether SCFI could differentiate between a cytotoxic and a non-cytotoxic drug, the Caco-2 colorectal cancer cell line was chosen, because it was previously reported that 10 $\upmu$M paclitaxel had little to no effect on Caco-2 cell viability^34^. The effects of staurosporine (10 $\upmu$M) and paclitaxel (10 $\upmu$M) on Caco-2 cells were therefore compared (Fig. 4).

Caco-2 cells were more sensitive to staurosporine than PC3 cells, evidenced by lower *p*-values when treated with staurosporine (Tables 1, 2), for both $g_{0}$ and $\tau _{D}$, compared to PC3 cells (Fig. 4). This is consistent with previously published research on the effects of staurosporine on the viability Caco-2 and PC3 cells^30,31,35–37^. No significant differences were observed between the last set of control and paclitaxel-treated cells (*p*-values = 0.37 and 0.07), confirming that Caco-2 cells are only minorly affected by the paclitaxel treatment, which is consistent with prior research as $IC_{50}$ for Caco-2 cells treated with paclitaxel for three entire days has been estimated to c. 10 $\upmu$M^34^, while at least an order of magnitude less for PC3 cells^32^. As such, the concentrations used in this experiment can arguably be considered clinically meaningful. Staurosporine, in contrast, has $IC_{50}$ values in the nM for most cell lines^38^. These results confirm that SCFI can discriminate between effective and non-effective treatments on a cancer cell line.

### Effects of cell confluency on SCFI measurements

The SCFI measurements described above for PC3 and Caco-2 cells were determined using single (PC3) and sub-confluent (Caco-2) cells. Epithelial cells such as Caco-2 cells, however, form E-cadherin-based cell-cell junctions and stop proliferating when they are confluent^39^. To test whether confluence and quiescence affect the level of fluctuations, SCFI measurements were carried out at 2, 4, and 7 days after plating Caco-2, Calu-3, and A549 cells. At 2 days, confluency was estimated at 60%, before achieving estimated 100% confluency by day 7.

To determine whether other epithelial cell lines behaved similarly to Caco-2 cells at different confluencies, Calu-3 (human epithelial lung cancer) and A549 (human epithelial alveolar basal cancer) cell lines were used. Similar to Caco-2 cells, these two cell lines grow optimally in colonies^40^. Both cell lines behaved similarly to Caco-2 cells during culture for 7 days.

**Fig. 5:**
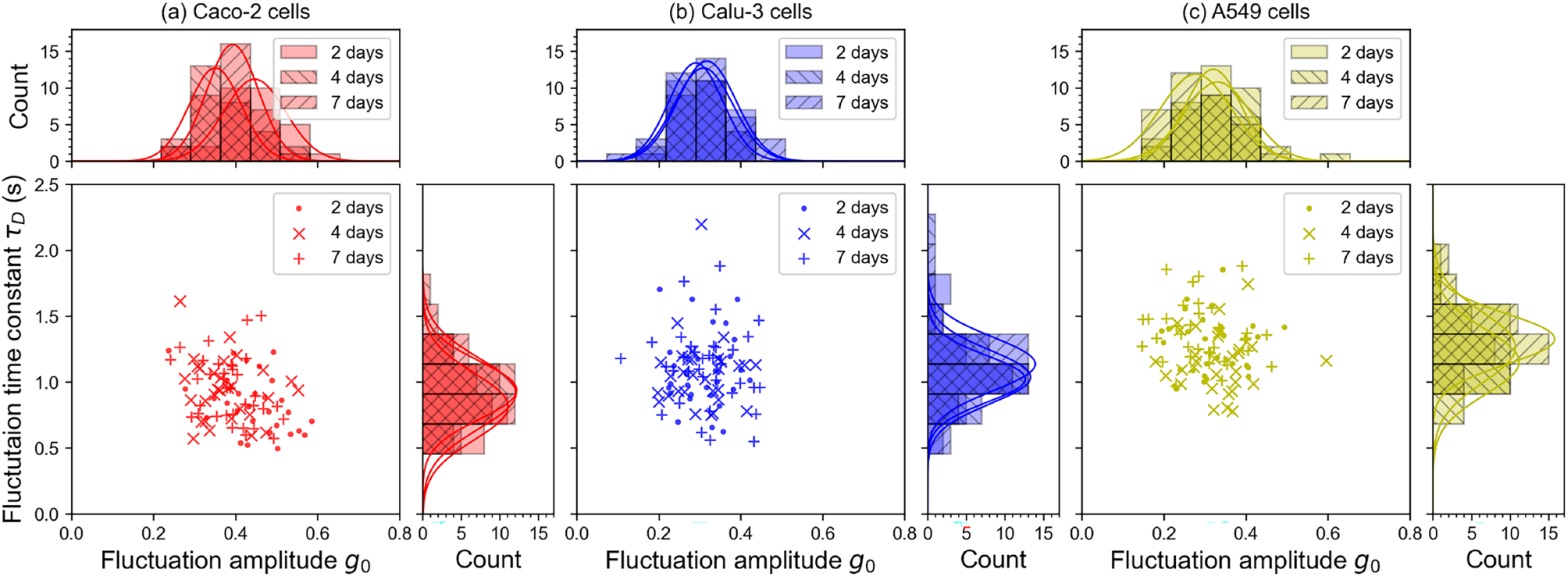
**SCFI fluctuation parameters are stable over time and independent of cell confluency.** To assess whether cell density influences SCFI measurements, three different epithelial cell lines - **(a)** Caco-2, **(b)** Calu-3, and **(c)** A549 (for each cell line n = 95) - were cultured for up to 7 days, progressing from sub-confluent (day 2) to fully confluent (day 7). For each cell line, the distributions of fluctuation amplitude ($g_{0}$) and time constant ($\tau _{D}$) show no statistically significant change over the 7-day period, demonstrating that the SCFI measurement is robust and independent of cell confluency. The data also reveal that each cell line has a distinct baseline fluctuation profile, with Caco-2 cells exhibiting the fastest fluctuations (lowest $\tau _{D}$) and A549 cells the slowest (highest $\tau _{D}$), suggesting that the technique is sensitive to inherent physiological differences between cell types (see “Methods”)

Examining each cell line over 2, 4 and 7 days, we generally detected no statistically significant differences ($p>0.05$) between different days for both $g_{0}$ and $\tau _{D}$ (see Fig. 5 and Table 3 below, and Table S1 in Supplementary Information). In a minority of cases, a statistically significant difference was observed for one of the two parameters, but we recorded no cases in which both parameters had a *p*-value $<0.05$. This suggests that SCFI fluctuations are independent of cell confluency.

These experiments also demonstrated that whilst the fluctuation parameters of untreated cells remained constant over time, untreated Caco-2 cells fluctuate slightly faster on average when compared to Calu-3 and A549 cell lines (0.91 vs. 1.09 and 1.27 s, respectively). The difference between the fluctuation time constants of three cell lines is statistically significant (see Table 3 below), so it is probably related to physiological differences between different cell lines. This result indicates the possibility of using SCFI not only to evaluate cytotoxic efficacy but also to characterise and potentially identify specific cell lines.

Considering the reproducibility and similarity between the SCFI data on PC3, Caco-2, Calu-3 and A549 cells and the fact that these cell types include a wide range of human cancer cell lines, it is likely that SCFI could be applied to many human cancer cell lines, both with regard to the quantitative fluctuation mean for untreated cells that do not experience environmental issues, but also in that there is no time-dependence nor dependency on confluency. This, in turn, implies that SCFI could be used to evaluate cytotoxic drug efficacy in most human cancer cell lines with a high level of reliability and consistency.

**Table 3:** Average parameters for Fig. 5, with mean *p*-values given for $g_{0}$ and $\tau _{D}$. For the full breakdown of all possible comparisons, see Table S1 in Supplementary Information

	Caco-2	Calu-3	A549
Combined $g_{0}$	0.401±0.008	0.312±0.007	0.311±0.008
Combined $\tau _{D}$	0.91±0.02 s	1.09±0.03 s	1.27±0.02 s
Mean *p*-val ($g_{0}$)	0.15	0.54	0.27
Mean *p*-val ($\tau _{D}$)	0.35	0.70	0.046

### SCFI is able to evaluate cytotoxicity within 3 h of cell plating

**Fig. 6:**
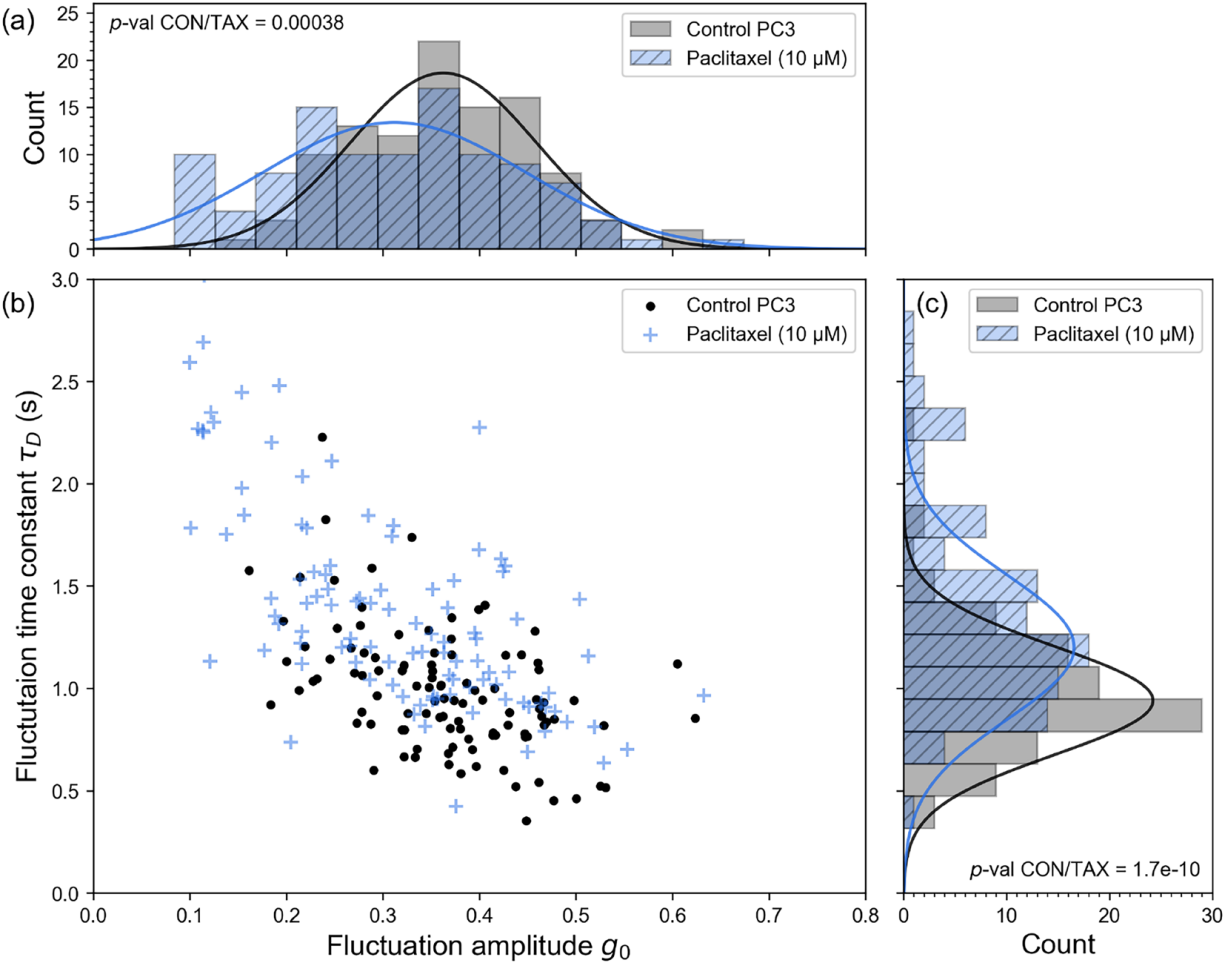
**SCFI can detect cytotoxic effects within hours of cell plating, eliminating the need for overnight incubation.** To test a faster workflow, PC3 cells were seeded and immediately treated with paclitaxel (10 $\mu$M) or a vehicle control (DMSO), with SCFI measurements starting approximately 3 h after plating. The data show a clear and statistically significant difference between the control and paclitaxel-treated groups for both fluctuation amplitude ($g_{0}$, shown in **(a, b)**) and fluctuation time constant ($\tau _{D}$, shown in **(b, c)**). This shows that SCFI can reliably assess drug efficacy on the same day the cells are prepared, greatly reducing the total assay time compared to traditional methods. Data are from 210 cells pooled from three independent experiments (105 control and 105 paclitaxel treated, each repeat 35 per set). See Table 4 for more data statistics

We have demonstrated that SCFI is able to evaluate cytotoxic drug efficacy reliably within 3 h after applying either staurosporine or paclitaxel, using cells plated on the previous day and incubated overnight (Figs. 3,4). This makes SCFI faster at detecting effects of treatments than other currently used methods such as MTT and LDH^4^. To investigate whether pre-plating of cells could be eliminated from the protocol in order to speed up the assay, PC3 cells were seeded in the prescence of paclitaxel or control vehicle (DMSO), approximately 3 h before SCFI. Mean fluctuations of untreated cells were similar to PC3 cells pre-plated one day before experiments (Fig. 6 vs Fig. 3). Detailed statistics for data presented in Fig. 6 are shown in Table 4.

Under these conditions, paclitaxel reduced the amplitude and speed of sub-cellular fluctuations, similar to pre-plated cells. The cells measured here had a similar appearance and shape to the pre-plated PC3 cells previously measured, but estimated lower confluency. Overall, these results demonstrate that SCFI is able to detect the effects of cytotoxic drugs rapidly and reliably within 3 h of addition, even to cells initially in suspension, which would be similar to cells isolated from human tumours.

**Table 4:** Detailed statistics for data presented in Fig. 6. Means, standard deviations and limits. For a detailed comparison of p-values, see Table S2 in Supplementary Information. CON, control; TAX; paclitaxel

PC3	$g_{0}$ mean	$g_{0}$ SD	$g_{0}$ lower	$g_{0}$ upper	$\tau _{D}$ mean	$\tau _{D}$ SD	$\tau _{D}$ lower	$\tau _{D}$ upper
CON	0.36	0.09	0.35	0.38	0.98	0.31	0.93	1.04
TAX	0.31	0.12	0.29	0.33	1.40	0.55	1.21	1.62

### SCFI results are validated using a MTT cell viability assay

To further validate the results acquired with SCFI using a widely used cell viability assay, we used MTT uptake assays. PC3 cells were seeded in 96-well plates following a similar protocol to the SCFI experiments. After 24 h, cells were treated with varying concentrations of paclitaxel or staurosporine for 24 h, which included concentrations used in SCFI experiments (Figure S2, Supplementary Information). Subsequently, MTT solution was added and cells incubated for 4 h. The relative MTT uptake by cells was reduced by both paclitaxel and staurosporine (Figure S2, Supplementary Information). These MTT results were consistent with the SCFI measurements.

## Discussion

Here we have demonstrated that SCFI can be used to identify responses of human cells to cytotoxic drugs. The speed, sensitivity and versatility of SCFI are amongst the main advantages of this technique when compared to currently available colorimetric or fluorescence-based assays for measuring effects of drug treatments (see Introduction). The method is also completely label-free and carried out on live cells, allowing responses to treatments to be monitored over time. We have shown that SCFI can be used to measure the effects of two different cytotoxic drugs quantitatively, and on multiple cancer cell lines, without having to wait for individual cell death or a reduction in cell number. By observing sub-cellular activities within single cells, SCFI also has the potential to provide insight into how a given drug behaves inside or at the boundaries of an individual cancer cell, and hence observe the variability of individual cells in a population in their response to drugs. We have also shown that the technique works reliably both on single cells (e.g. PC3) and on cells in large groups (e.g. Caco-2). We have demonstrated that a wide range of different human cancer cell lines can be used with SCFI, which implies that the technique could be used for antineoplastic efficacy testing in most cancer cell lines.

Interestingly, we have found that SCFI is able to produce statistically significant results within 3 h of applying an antineoplastic drug, even without overnight incubation. This means that our technique is significantly faster than most, if not all, currently used alternatives^4^. The acquisition of results that are statistically similar from different SCFI microscopes operating on the same principles also demonstrates that the technique possesses a high degree of reliability and consistency, which would be required for clinical use^21^. Furthermore, the time-autocorrelation analysis is robust and effective, producing two independent metrics of cell viability. When considering trials with cytotoxic drugs, either the fluctuation amplitude $g_{0}$ or the fluctuation time constant $\tau _{D}$ could be used in isolation to identify an effective treatment positively. Consideration of both parameters together allows researchers to study many aspects of cellular responses to treatment in real time.

More research on SCFI applications will be necessary before it can be developed for potential clinical applications, but the initial results described here are very promising, and the overall method has been demonstrated to work well to identify early responses of cells to cytotoxic drugs. The quantification of antineoplastic efficacy of additional clinically relevant drugs and cancer-derived patient cells necessary for clinical screening will require further research. It would also be important to test if the technique is able to generate usable fluctuation measurements from cells taken directly from cancer patients; this should work in theory and will likely increase the reliability of cell-based testing when compared to labour-intensive in vivo investigations using animals. SCFI therefore presents the possibility of a quick, reliable, and label-free way to test cytotoxic efficacy.

It would be interesting to compare cancer and non-cancer cells from the same cell type or patient using SCFI, which would help determine if the method could be used for diagnostic purposes. Given that recent research has demonstrated differences in cellular deformability between cancerous and non-cancerous cells^41^, and that the resulting change in cytoplasmic viscosity should affect fluctuation magnitudes of sub-cellular objects^42^, it is quite possible that SCFI measurements would differ between normal and cancer cells.

While the results presented here are promising, we acknowledge some critical limitations. The current SCFI system relies on specialised optical instrumentation, and its accessibility for widespread clinical use will depend on future development, such as the lensless platform we mention later. This study serves as a proof-of-principle, and before SCFI can be applied in clinical oncology, extensive validation on a broader panel of drugs and both cancerous and non-cancerous cell lines is required. Further work on primary cells from patient biopsies is also a critical next step. Finally, while our data seem to be consistent with the hypothesis that fluctuations are somehow linked to cellular metabolism, the precise biological origin of these signals warrants further investigation.

A well-characterised MTT cell viability assay confirmed the SCFI results, which were also consistent with previously published work^30–32,34–37^. While this is sufficient to demonstrate that the SCFI method works, future studies would benefit from benchmarking against a wider array of assays. The primary focus of this work, however, is the development of a new rapid-testing modality. Beyond oncology, the ability to rapidly assess cellular viability has broad applications in fields such as infectious disease for antimicrobial susceptibility testing, and in pharmacology for general toxicity screening. To address the need for higher throughput, for example, a lensless SCFI platform is in development. This approach replaces the complex microscope optics with a simplified system where a multi-well plate can be analysed directly over a detector^21^. This design enables parallel, automated measurements from multiple samples, providing a clear roadmap for scaling the technology for cost-effective, high-throughput drug screening.

The most significant potential of this scalable technology lies in its application to personalised medicine. The ability to rapidly test a panel of drugs on primary cells taken directly from a patient could enable clinicians to identify the most effective treatment within a clinically relevant timeframe. Translating SCFI to the clinic will require addressing several practical challenges, including sample handling, cell viability, and tumour heterogeneity. Our demonstration that the assay works on cells plated just three hours prior to measurement is crucial, as it aligns with the limited viability window of primary cells isolated from biopsies. Standard protocols for enzymatic and mechanical dissociation of tumour tissue can yield single-cell suspensions within 1-2 hours, making a same-day ’sample-to-answer’ workflow feasible. Furthermore, because SCFI is a single-cell technique, it can generate a distribution of drug responses from across a cell propulation, potentially revealing resistant subpopulations that bulk assays would miss.

In conclusion, we have established SCFI as a rapid, sensitive, and label-free method for evaluating the efficacy of cytotoxic drugs. Its robustness across various cancer cell lines and independence from cell confluency highlight its broad applicability. By providing single-cell data within hours, SCFI presents a promising new tool to accelerate the move towards truly personalised cancer treatment, reducing patient exposure to ineffective therapies.

## Methods

### Cell culture and treatments

PC3 cells were grown in RPMI (Gibco) containing 10% FBS and 1% Pen-Strep (Gibco) to a confluency of >70% before the cells were incubated with Trypsin-EDTA (Thermo Fisher Scientific), centrifuged, resuspended, counted, and then seeded for experiments. For all tests involving PC3 cells (apart from the ‘same-day’ tests), $\text{3 }\times \text{10 }^4$ cells were seeded into an Ibidi 35 mm $\mu$-dish (polymer coverslip) in 2 ml of growth medium, one day before measurements were taken, and incubated overnight (18-24 h). All incubations undertaken at ${37}^\circ C$ unless stated otherwise. After overnight incubation, the medium was exchanged to 2 ml L-15 medium (Gibco), which is buffered to air $\text{ CO}_2$ levels because the incubator neighbouring the SCFI microscope did not have $\text{ CO}_2$ control. staurosporine (Selleckchem), paclitaxel (Stratech), or the same volume of control vehicle (DMSO, Gibco) was mixed with the L-15 medium prior to adding it to the petri dish. The samples were then transported to the SCFI microscopy laboratory in a sealed and insulated container, and incubated for approximately 30-40 min before the first set of measurements were acquired. For cells analysed on the same day as seeding, cells were trypsinised, centrifuged, and resuspended in L-15 medium containing 10 $\mu$M paclitaxel or the same volume of DMSO, then transported and subsequently incubated for approximately 2.5 h (otherwise the same protocol as for the other PC3 samples).

Caco-2 and A549 cells were maintained in DMEM (Gibco) containing 10% FBS, 1% Pen-Strep (Gibco), and 1% 200 mM L-glutamine (Gibco), which was also used during measurements due to the availability of an incubator with $\text{ CO}_2$ control. Calu-3 cells were maintained in MEM (Gibco) containing 10% FBS, 1% Pen-Strep (Gibco), 1% 200 mM L-glutamine (Gibco), 1% 100 mM sodium pyruvate (Gibco), and 1% non-essential amino acids (Gibco), which was also used during measurements. Cells ($\text{5 }\times \text{10 }^4$) were seeded on 35 mm culture dishes (Greiner Cellview non-treated glass bottom). After overnight incubation and prior to SCFI measurements, cells were given new fresh growth medium.

PC3 cells were provided by Dr Magali Williamson (King’s College London) and authenticated by Eurofins. Caco-2 (HTB-37), A549 (CCL-185), and Calu-3 (HTB-55) cells were sourced from ATCC. The Calu-3 cells were not revalidated.

#### MTT assay

PC3 cells ($2\times 10^4$) were plated in 96-well plates in 100 $\mu$L RPMI with 10% FBS. After 24 h, paclitaxel or staurosporine (concentrations between 0 and 5 $\mu$M) were added for 24 h in technical triplicate wells, followed by addition of 10 $\mu$L of MTT solution (Biotium) per well. Cells were incubated at ${37}^\circ C$ for 4 h, then 200 $\mu$L DMSO was added. The absorbance at 595 nm (absorbance) and 655 nm (background absorbance) were measured and the background absorbance subtracted from the absorbance to obtain normalized absorbance values. Additional concentration tested derived from 2-fold serial dilutions.

#### SCFI instrumentation

The SCFI instrumentation has previously been described with more detailed information in a paper currently in pre-print by Bermingham *et al.*, which also covers the technical aspects of SCFI in greater detail^21^.

The SCFI method is completely label-free, and capable of high-resolution detection and real-time imaging of light scattered by sub-cellular fluctuations inside a single cell. The SEW is generated using a laser and a high numerical aperture (NA) objective lens. Total internal reflection of the laser beam within the lens (Fig. 1) creates an evanescent field wich penetrates approximately 100 nm into the sample. To adjust focal position, sample movement in the vertical direction is provided by a piezo inertia actuator. The beam reflected from the surface is safely deflected without disturbing the optical detection system. The SEW is collected using the high NA objective, after which it is reflected and then directed to a high-resolution CMOS camera via a tube lens. Movement in the horizontal direction is facilitated by an x-y slip-stick positioning stage, which is capable of positioning objects with sub-micron accuracy inside the evanescent field.

Two systems with slightly different components were used for imaging, although both worked on identical principles and functioned in near-identical ways. The first system was used for all measurements of PC3 cells, and uses the following components:

1. Hamamatsu Orca4 sCMOS (pixel size 6.5$\times$6.5 $\upmu$m, quantum efficiency 70% at 561 nm, RMS readout noise 1.5 $e^{-1}$).
2. 561 nm fibre-coupled diode laser (Vortran Stradus), with a beam diameter post collimation of 1 $\upmu$m, and typical power after the fibre of 1 mW.
3. Nikon TIRF 1.49 NA objective lens.
4. Image size on video camera sensor of 26.5 nm/pixel.

The second system was used for all measurements of Caco-2, Calu-3 and A549 cells, and uses the following components:

1. Thorlabs Quantalux sCMOS (pixel size 5.04$\times$5.04 $\upmu$m, quantum efficiency 55% at 488 nm, RMS readout noise <1.5 $e^{-1}$).
2. 488 nm fibre-coupled diode laser (Vortran Stradus), with a beam diameter post collimation of 5 $\upmu$m, and typical power after the fibre of 30 mW.
3. Nikon TIRF 1.49 NA objective lens.
4. Image size on video camera sensor of 29 nm/pixel.

Measurement of time constant ($\tau _{D}$) depends not just on the speed within an observation volume, but also on the effective area of the sensor used^27,29^. For SCFI systems, each pixel is considered a sensor, and the effective detection area is related directly to the point-spread function of the microscope as well as the level of magnification. Since system 1 and system 2 had both different illuminating wavelengths, as well as different magnification levels, it would be incorrect to directly compare measurements from system 1 against system 2.

#### Image acquisition and analysis

A dish without cells was used to focus the microscope approximately. Immediately before taking measurements, cells were placed on the SCFI microscope and the focus adjusted as required. Measured cells were positioned in the centre of the evanescent field, and only cells deemed from bright-field observation to be fully attached to the surface were measured. For the PC3 cells, only cells that were not located right next to other cells were measured (minimum distance of one cell diameter between cells in each direction was enforced). For the other cell lines, measured cells were in colonies of minimum 10 cells, and fully surrounded by other cells. Between 20 to 35 cells were measured during each session, and cells were returned to an incubator after a maximum exposure of approximately 45 min. All measurements in a given session were completed before the results were analysed.

SCFI produces a 300$\times$300 pixel monochromatic frame sequence for each cell, corresponding to an area within the cell of approximately 56 $\upmu$m$^2$ (see Fig. 2). Data were taken at 20 frames per second over 20 s, resulting in a 400-frame sequence. To speed up computation, we fixed a region of interest (ROI) throughout the recorded frame sequence for analysis. The ROI was 30$\times$30 pixels with an actual size of approximately 1.08 $\upmu$m$^2$. Preliminary analysis showed a consistent convergence of results for ROI sizes $\ge$ 20$\times$20 pixels, i.e., including the entire movie served only to increase computation time. Finally, both the ROI, and the larger video area, were completely contained within the region of the cell, which was in turn contained within the evanescent field as described above.

The data analysis computes a time-autocorrelation function (ACF) at the pixel level for a SCFI recoded frame sequence^27,43^. To calculate the ACF, the following equation^27^ is applied to the intensity time trace *I*(*t*) of each pixel:

1 $$\begin{aligned} { G_{p}(\tau ) = \frac{\left\langle \delta I(t) \cdot \delta I(t + \tau ) \right\rangle }{\left\langle I - I_{B}\right\rangle ^{2}} } \end{aligned}$$

**Fig. 7:**
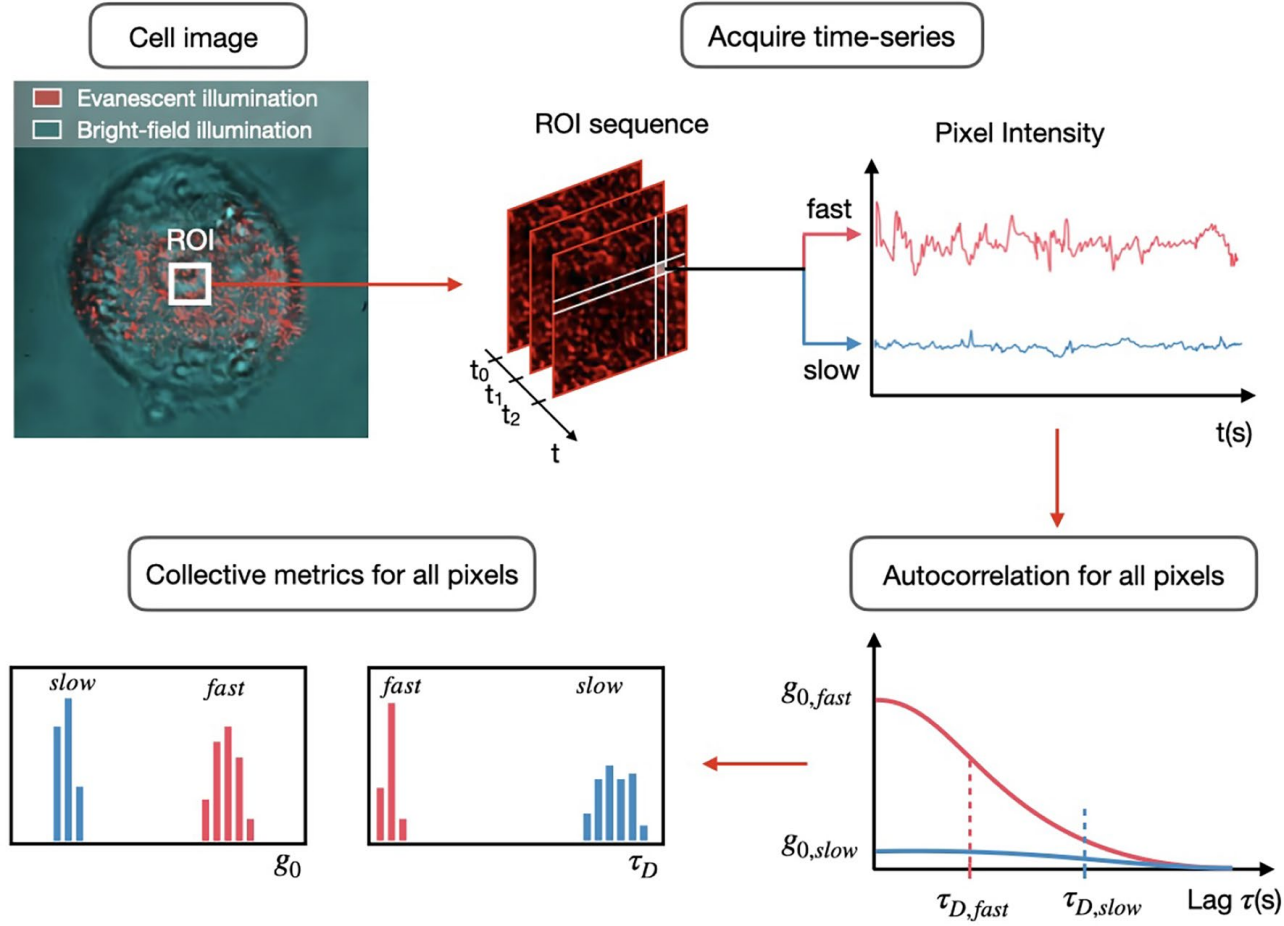
**Schematic of the SCFI data analysis workflow.** This workflow illustrates how raw imaging data are processed to produce quantitative metrics of subcellular activity. First, a time-series of evanescent field scattering images is acquired from a Region of Interest (ROI) within a single cell. The intensity fluctuations over time are recorded for each individual pixel. An autocorrelation analysis is then performed on each pixel’s intensity trace, resulting in a curve whose shape is characteristic of the underlying motion. From a fit to this curve, two key parameters are derived: the fluctuation amplitude ($g_{0}$), which relates to the magnitude of motion, and the fluctuation time constant ($\tau _{D}$), which relates to the velocity. This is illustrated for two theoretical cells with “fast” (red) and “slow” (blue) internal dynamics. Finally, the values of $g_{0}$ and $\tau _{D}$ from all pixels in the ROI are compiled into histograms, and the median of each distribution is used as the final representative metric for that cell

Here $I_{B}$ denotes minimum background intensity across all pixels. The mean intensity $\left\langle I \right\rangle$ is subtracted from the original signal *I*(*t*) to obtain the fluctuation from the mean $\delta I(t)$. A normalisation factor $\left\langle I - I_{B} \right\rangle ^{2}$ ensures that the ACF amplitude does not depend on the illumination intensity. $G_{p}(\tau )$ is fitted using an exponential function (Eq. 2) that provides two fitting parameters: the time constant $\tau _{D,p}$, which quantifies the time-scale of the signal *I*(*t*), and the fluctuation amplitude $g_{0,p}$ which represents the amplitude of fluctuation and is related to the variance of the pixel intensity^29^. More appropriate models based on the physical theory behind the fluctuations can be use to fit the ACF^28^. However, as a differentiating tool, the single exponential decay is robust and, as we have seen, sufficient to differentiate between different cell states. Figure 7 shows each step in the ACF analysis, for both an active and inactive PC3 cell.

2 $$\begin{aligned} { G_p^*=g_{0,p}\exp ({-\tau /\tau _{D,p}}) } \end{aligned}$$

The ACF analysis of a cell produces two distributions of values: one for the time-scale $\tau _{D,p}$, and a second for the fluctuation amplitude $g_{0,p}$ of recorded sub-cellular processes. The number of points (*p*) in each distribution is given by the number of pixels in the selected ROI, in this case 900 pixels. By calculating the median of each distribution, we obtain a pair of values ($\tau _{D}$ and $g_{0}$) associated with the particular cell. This process is repeated for different cells to obtain a representative sampling of the original cell culture. When comparing the treated and control samples, $\tau _{D}$ and $g_{0}$ distributions are compared separately.

Processing, data analysis and figure plotting were all performed using Python 3.10. To load each video, compute and fit the ACF to each pixel, and save the resultant arrays typically took approximately 10 minutes for a set of 50 measurements when cropped to a 30$\times$30 ROI. To perform the same operation on the whole frame (i.e. without cropping) took approximately 40 minutes per video. These figures are accurate with respect to a standard spec laptop computer (16GB Apple M1 Pro).

#### Statistical analysis of the fluctuation distributions

To determine the statistical significance of whether or not individual fluctuation data sets differ from each other, Welch’s two-sided t-test for two independent samples (assuming unequal variance) was used to determine the t-statistic and *p*-value. This was used due to its simplicity and the limited number of single or pooled data sets compared in each individual experiment. The statistical difference to reject the null hypothesis was assumed to be *p* = 0.05. Relevant *p*-values not provided in figures or main text can be found in the Supplementary Information. Furthermore, Lehr’s equation is commonly used for estimation of required sample sizes^44^. For all statistically significant comparisons (*p* < 0.05), the minimum required sample size through Lehr’s equation was less than the actual size of the sampled datasets, using standard false positive and false negative probabilities of 0.05 and 0.2 respectively.

## Supplementary Information

Supplementary Information.

## Data Availability

All data generated in this research are stored on the University of Bristol research data storage facility, available from the corresponding author on reasonable request.

## References

[1] Begley, C. G. & Ellis, L. M. Raise standards for preclinical cancer research. *Nature* **483**, 531–533 (2012). 22460880 10.1038/483531a

[2] Lopez-Lazaro, M. A simple and reliable approach for assessing anticancer activity in vitro. *Curr. Med. Chem.* **22**, 1324–1334 (2015). 25666807 10.2174/0929867322666150209150639

[3] Niles, A. L., Moravec, R. A. & Riss, T. L. In vitro viability and cytotoxicity testing and same-well multi-parametric combinations for high throughput screening. *Curr. Chem. Genomics* **3**, 33–41 (2009). 20161834 10.2174/1875397300903010033 PMC2802765

[4] Fotakis, G. & Timbrell, J. A. In vitro cytotoxicity assays: Comparison of LDH, neutral red, MTT and protein assay in hepatoma cell lines following exposure to cadmium chloride. *Toxicol. Lett.* **160**, 171–177 (2005). 16111842 10.1016/j.toxlet.2005.07.001

[5] Vermes, I. et al. A novel assay for apoptosis flow cytometric detection of phosphatidylserine expression on early apoptotic cells using fluorescein labelled annexin V. *J. Immunol. Methods* **184**, 39–51 (1995). 7622868 10.1016/0022-1759(95)00072-i

[6] Kim, Y. et al. Fluorometric assay of DNA in cartilage explants using Hoechst 33258. *Anal. Biochem.* **174**, 168–176 (1988). 2464289 10.1016/0003-2697(88)90532-5

[7] McMaster, G. & Carmichael, G. Analysis of single and double-stranded nucleic acids on polyacrylamide and agarose gels by using glyoxal and acridine orange. *PNAS* **74**, 4835–4838 (1977). 73185 10.1073/pnas.74.11.4835 PMC432050

[8] Nicoletti, I. et al. A rapid and simple method for measuring thymocyte apoptosis by propidium iodide staining and flow cytometry. *J. Immunol. Methods* **193**, 271–279 (1991). 10.1016/0022-1759(91)90198-o 1710634

[9] Hatzis, C. et al. Enhancing reproducibility in cancer drug screening: How do we move forward?. *Cancer Res.* **74**, 4016–4023 (2014). 25015668 10.1158/0008-5472.CAN-14-0725 PMC4119520

[10] Pelling, A. E., Sehati, S., Gralla, E. B., Valentine, E. S. & Gimzewski, J. K. Local nanomechanical motion of the cell wall of Saccharomyces cerevisiae. *Science* **305**, 1147–1150 (2004). 15326353 10.1126/science.1097640

[11] Kasas, S. et al. Detecting nanoscale vibrations as signature of life. *Proc. Natl. Acad. Sci.* **112**, 378–381 (2015). 25548177 10.1073/pnas.1415348112 PMC4299216

[12] Longo, G. et al. Rapid detection of bacterial resistance to antibiotics using AFM cantilevers as nanomechanical sensors. *Nat. Nanotechnol.* **8**, 522–526 (2013). 23812189 10.1038/nnano.2013.120

[13] Aghayee, S. et al. Combination of fluorescence microscopy and nanomotion detection to characterize bacteria. *J. Mol. Recognit.* **26**, 590–595 (2013). 24089366 10.1002/jmr.2306

[14] Lissandrello, C. et al. Nanomechanical motion of Escherichia coli adhered to a surface. *Appl. Phys. Lett.* **105**, 113701 (2014). 25316924 10.1063/1.4895132 PMC4187256

[15] Etayahi, H., Khan, M. F., Kaur, K. & Thundat, T. Microfluidic cantilever detects bacteria and measures their susceptibility to antibiotics in small confined volumes. *Nat. Commun.* **7**, 12947 (2016). 27698375 10.1038/ncomms12947 PMC5059454

[16] Syal, K. et al. Plasmonic imaging of protein interactions with single bacterial cells. *Biosens. Bioelectron.* **63**, 131–137 (2015). 25064821 10.1016/j.bios.2014.06.069

[17] Syal, K. et al. Antimicrobial susceptibility test with plasmonic imaging and tracking of single bacterial motions on nanometer scale. *ACS Nano* **10**, 845–852 (2015). 26637243 10.1021/acsnano.5b05944

[18] Kara, V. et al. Microfluidic detection of movements of Escherichia coli for rapid antibiotic susceptibility testing. *Lab Chip* **18**, 743–753 (2018). 29387860 10.1039/c7lc01019b PMC5829026

[19] Johnson, W. L., France, D. C., Rentz, N. S., Cordell, W. T. & Walls, F. L. Sensing bacterial vibrations and early response to antibiotics with phase noise of a resonant crystal. *Sci. Rep.* **7**, 12138 (2017). 28939857 10.1038/s41598-017-12063-6 PMC5610186

[20] Guliy, O. I., Zaitsev, B. D. & Borodina, I. A. New approach for determination of antimicrobial susceptibility to antibiotics by an acoustic sensor. *Appl. Microbiol. Biotechnol.* **104**, 1283–1290 (2020). 31865437 10.1007/s00253-019-10295-2

[21] Bermingham, C. R. et al. Imaging of sub-cellular fluctuations provides a rapid way to observe bacterial viability and response to antibiotics. *Sensors* (preprint at bioRxiv.org). TBC. 10.1101/460139 (2025).

[22] Jorgensen, J. H. & Ferraro, M. J. Antimicrobial susceptibility testing - A review of general principles and contemporary practices. *Clin. Infect. Dis.* **11**, 1749–1755 (2009). 10.1086/647952 19857164

[23] Okeke, I. N. et al. Diagnostics as essential tools for containing antibacterial resistance. *Drug Resist. Updates* **14**, 95–106 (2011). 10.1016/j.drup.2011.02.002 21398170

[24] Apelian, C. et al. Dynamic full field optical coherence tomography: subcellular metabolic contrast revealed in tissues by interferometric signals temporal analysis. *Biomed. Opt. Exp.* **7**, 1511–1524 (2016). 10.1364/BOE.7.001511 PMC4929658 27446672

[25] Antognozzi, M. et al. A new detection system for extremely small vertically mounted cantilevers. *Nanotechnology* **19**, 384002 (2008). 21832562 10.1088/0957-4484/19/38/384002

[26] Antognozzi, M. et al. Direct measurements of the extraordinary optical momentum and traverse spin-dependent force using a nano-cantilever. *Nat. Phys.* **12**, 731–735 (2016).

[27] Krieger, J. W. et al. Imaging fluorescence (cross-) correlation spectroscopy in live cells and organisms. *Nat. Protoc.* **10**, 1948–1974 (2015). 26540588 10.1038/nprot.2015.100

[28] Kolin, D. & Wiseman, P. Advances in image correlation spectroscopy: Measuring number densities, aggregation states, and dynamics of fluorescently labeled macromolecules in cells. *Cell Biochem. Biophys.* **49**, 141–164 (2007). 17952641 10.1007/s12013-007-9000-5

[29] Wiseman, P. W. Image correlation spectroscopy: Principles and applications. *Cold Spring Harbor Protoc.* **2015**, 336–348 (2015). 10.1101/pdb.top086124 25834267

[30] Li, X. Y. et al. Overexpression of BCL-X-L underlies the molecular basis for resistance to staurosporine-induced apoptosis in PC-3 cells. *Cancer Res.* **61**, 1699–1706 (2001). 11245486

[31] Nutt, L. K. et al. Bax-mediated Ca2+ mobilization promotes cytochrome c release during apoptosis. *J. Biol. Chem.* **23**, 20301–20308 (2002). 10.1074/jbc.M201604200 11909872

[32] Desai, N. et al. Increased antitumoractivity, intratumor paclitaxel concentrations, and endothelial cell transport of cremophor-free, albumin-bound paclitaxel, ABI-007, compared with cremophor-based paclitaxel. *Clin. Cancer Res.* **12**, 1317–1324 (2006). 16489089 10.1158/1078-0432.CCR-05-1634

[33] Mekhail, T. & Markman, M. Paclitaxel in cancer therapy. *Expert Opin. Pharmacother.* **3**, 755–766 (2002). 12036415 10.1517/14656566.3.6.755

[34] Sheppard, B. C. et al. Effects of paclitaxel on the growth of normal, polyposis, and cancerous human colonic epithelial cells. *Cancer* **85**, 1454–1464 (1999). 10193934

[35] Bissonnette, M. et al. 1.25(OH)2 vitamin D3 activates PKC- in Caco-2 cells. *Am. J. Physiol.* **267**, 465–475 (1994). 10.1152/ajpgi.1994.267.3.G465 7943245

[36] Basson, M. D. & Hong, F. Modulation of human Caco-2 intestinal epithelian cell phenotype by protein kinase C inhibitors. *Cell Biol. Int.* **19**, 1025–1032 (1995). 9721628 10.1006/cbir.1995.1045

[37] Chakrabarti, G., Zhou, X. & McClane, B. A. Death pathways activated in Caco-2 cells by Clostridium perfringens enterotoxin. *Infect. Immun.* **71**, 4260–4270 (2003). 12874301 10.1128/IAI.71.8.4260-4270.2003 PMC166005

[38] Tamaoki, T. et al. Staurosporine, a potent inhibitor of phospholipid/Ca++ dependent protein kinase. *Biochem. Biophys. Res. Commun.* **135**, 397–402 (1986). 3457562 10.1016/0006-291x(86)90008-2

[39] Kovacs, E. M. et al. N-WASP regulates the epithelial junctional actin cytoskeleton through a non-canonical post-nucleation pathway. *Nat. Cell Biol.* **13**, 934–944 (2011). 21785420 10.1038/ncb2290

[40] Knobel, P. A., Kotov, I. N., Felley-Bosco, E., Stahel, R. A. & Marti, T. M. Inhibition of REV3 expression induces persistent DNA damage and growth arrest in cancer cells. *Neoplasia* **13**, 961–970 (2011). 22028621 10.1593/neo.11828 PMC3201572

[41] Guck, J. et al. Optical deformability as an inherent cell marker for testing malignant transformation and metastatic competence. *Biophys. J.* **88**, 3689–3698 (2005). 15722433 10.1529/biophysj.104.045476 PMC1305515

[42] Lekka, M. et al. Elasticity of normal and cancerous human bladder cells studied by scanning force microscopy. *Eur. Biophys. J.* **28**, 312–316 (1999). 10394623 10.1007/s002490050213

[43] Bacia, K., Haustein, E. & Schwille, P. Fluorescence correlation spectroscopy: Principles and applications. *Cold Spring Harbor Protoc.* **2014**, 709–725 (2014) (Publisher: Cold Spring Harbor Laboratory Press). 10.1101/pdb.top081802 24987147

[44] Allen, J. Sample size calculation for two independent groups: A useful rule of thumb. *Stat. Proc. Singap. Healthc.* **20**, 138–140 (2011).

