# Supplementary material for: A rapid method of evaluating cytotoxic drug efficacy using sub-cellular fluctuation imaging
Source: Sci Rep. 2025 Dec 6;16:723. doi: 10.1038/s41598-025-30295-9 (PMC12780189; doi:10.1038/s41598-025-30295-9)
Supplement: Supplementary file 1 — Supplementary Information. [file 41598_2025_30295_MOESM1_ESM.pdf]

Supplementary information

Relevant supplementary data can be found below.

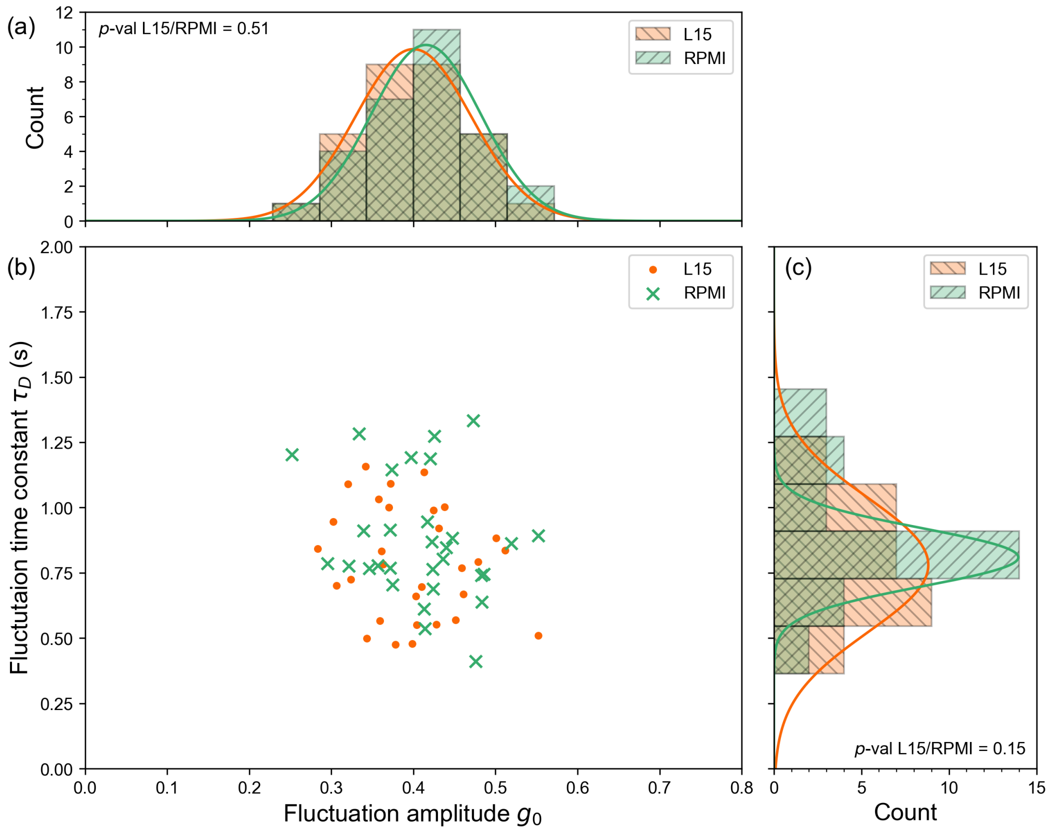

Figure S1. No statistical difference between effect of L15 and RPMI on PC3 cells. Measurements on 60 prostate cancer PC3 cells (30 changed from RPMI to fresh RPMI, 30 changed from RPMI to L-15; both *c.* 30 min prior to data acquisition). Single experiment. This is the only data set using PC3 cells that was measured using the second SCFI microscope system. The data are from one experiment. **(a)** shows a histogram of fluctuation amplitude g_0, **(b)** shows the intersection between fluctuation amplitude g_0 and fluctuation time constant τ_D, and **(c)** shows a histogram of fluctuation time constant τ_D. A p-value of 0.51 for g_0 distributions and 0.15 for τ_D distributions implies that changing the medium from RPMI to L-15 does not have a measurable effect on fluctuation parameters.

	**Caco-2**		
g_0	2d	4d	7d
2d	-	0.0012	0.0045
4d	0.0012	-	0.44
7d	0.0045	0.44	-

	**Calu-3**		
g_0	2d	4d	7d
2d	-	0.55	0.70
4d	0.55	-	0.36
7d	0.70	0.36	-

	**A549**		
g_0	2d	4d	7d
2d	-	0.44	0.29
4d	0.44	-	0.077
7d	0.29	0.077	-

τ_D	2d	4d	7d
2d	-	0.074	0.053
4d	0.074	-	0.91
7d	0.053	0.91	-

τ_D	2d	4d	7d
2d	-	0.87	0.67
4d	0.87	-	0.56
7d	0.67	0.56	-

τ_D	2d	4d	7d
2d	-	0.039	0.10
4d	0.039	-	0.00033
7d	0.10	0.00033	-

Table S1. p-values for g_0 and τ_D distributions shown in Fig. 5 within the main text. Measurements are expected to give $p > 0.05$ if fluctuations are independent of cell confluency. There are four failures: Caco-2 comparing the g_0 distributions for days 2/4 and 2/7, and A549 comparing the τ_D distributions for days 2/4 and 4/7. In all cases the other parameter gives $p > 0.05$. For any of the three cell lines, there are no comparisons across days where both g_0 and τ_D indicate statistically significant differences.

g_0	R1T	R2T	R3T	C1T	C2T	C3T
R1T	-	0.66	0.21	0.29	0.27	0.029
R2T	0.66	-	0.38	0.12	0.10	0.0052
R3T	0.21	0.38	-	0.022	0.015	0.00055
C1T	0.29	0.12	0.022	-	0.95	0.30
C2T	0.27	0.10	0.015	0.95	-	0.20
C3T	0.029	0.0052	0.00055	0.30	0.20	-

τ_D	R1T	R2T	R3T	C1T	C2T	C3T
R1T	-	0.99	0.11	0.0042	0.0048	0.00003
R2T	0.99	-	0.10	0.0033	0.0037	0.000018
R3T	0.11	0.10	-	0.00007	0.00008	9.1E-07
C1T	0.0042	0.0033	0.00007	-	0.77	0.072
C2T	0.0048	0.0037	0.00008	0.77	-	0.02
C3T	0.00003	0.000018	9.1E-07	0.072	0.02	-

Table S2. p-values for same-day seeding tests, shown in Fig. 6 within the main text. Rn stands for repeat n, T for treated sample, and C for control or untreated sample. The result is expected if $p > 0.05$ for T/T and C/C, or $p < 0.05$ for C/T and T/C combinations. Whilst there are cases where either g_0 or τ_D fails, there are no cases where g_0 and τ_D both produce the unexpected result.

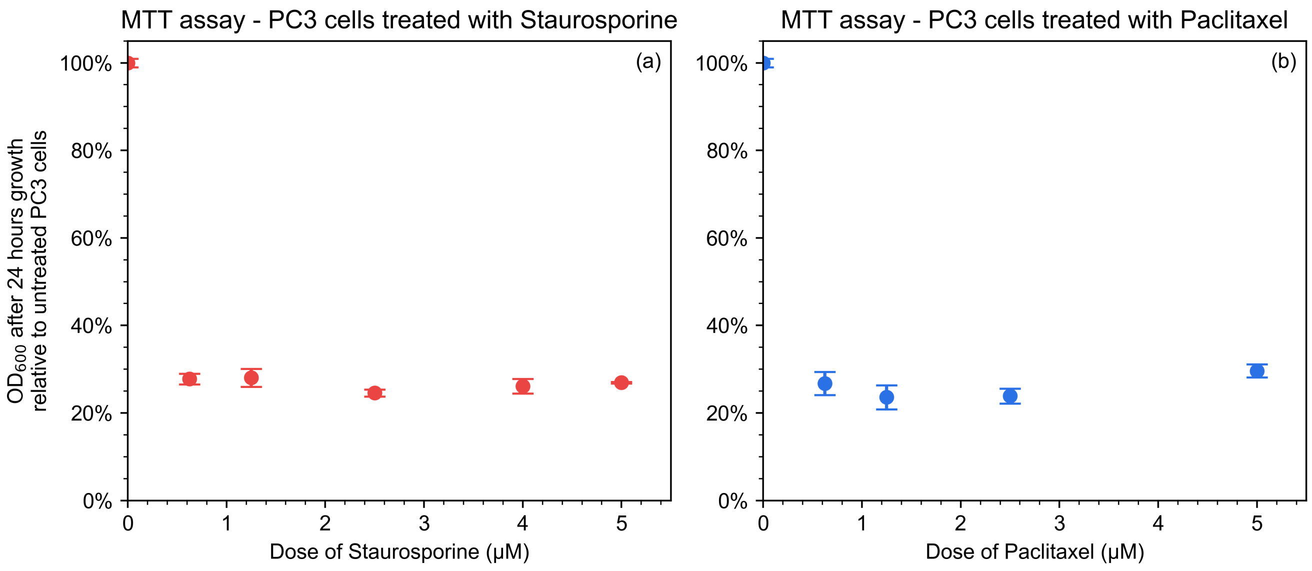

Figure S2. Results from staurosporine and paclitaxel on PC3 cells using MTT assay are consistent with SCFI. PC3 cells were seeded in 96-well plates following a similar protocol to the SCFI experiments, and after 24 hours the indicated concentrations of **(a)** staurosporine and **(b)** paclitexel were added for 24 hours. MTT solution was then added and cells incubated for 4 hours, then the MTT was dissolved in DMSO. The relative MTT uptake compared to control untreated PC3 cells is shown. Values show means and standard deviations.
